# Death While under Ether

**Published:** 1877-08

**Authors:** 


### ARTICLE VIII.

### *Death while under Ether at the London Hospital.*

An old man, sixty-nine years of age, was admitted into the London Hospital on May 12, suffering with obstruction of the bowels. He appeared old, even for his age. He was in considerable pain, which was referred chiefly to the right iliac region, and there was some tenderness also in the same place, as well as over the rest of the abdomen, which was distended and tense. It appeared from the history that the symptoms dated from two days previously ; he was the subject of hernia, which came down from time to time, although he wore a truss. Two days before, his hernia being down, he reduced it, the reduction causing pain. He had been sick previous to the reduction, and the vomiting and prevailed afterwards. The surgeon to the case diagnosed a reduction *en masse*, both from the history and also the appearance of a depression in the integument over the external ring ; and it was thought that some portion of an old sac, which was adherent to this integument had been reduced, and was dragging upon the integument and so giving rise to the depression or dimpling of the soft parts. With a view to get the hernia down, and to obviate the struggling which the pain of this process gave rise to, ether was administered. Clover's inhaler was used. For about thirty seconds he inspired only his expired air ; he, then had, for about a minute, one-quarter to a half ether. There was some struggling at the commencement, and he did not breathe in the ether well. The mouthpiece was therefore frequently removed from his face. The amount of ether was then diminished, and as his lips looked blue it was entirely stopped ; his breathing improved a little, but was not quite satisfactory. His pulse gradually became weaker, and finally stopped ; respiration, however, continuing for thirty seconds or more. Sylvester's method of artificial respiration and galvanism were resorted to without success. The post-mortem examination showed that a large coil of small intestine had got caught in a figure-ofeight-shaped loop of mesentery adherent by both ends to peritoneum under the above mentioned pouch. The intestine was very much inflamed, and of a deep purple colour. Left ventricle uncontracted and flaccid. Lungs: Extreme emphysema, and containimg little blood. Slight bronchitis. It would seem as though the man died more of shock than of ether. The jury also held this view, and returned, "Death from natural causes."—*Med. Times and Gaz.*

---

